# Establishing an optimized ATAC-seq protocol for the maize

**DOI:** 10.3389/fpls.2024.1370618

**Published:** 2024-05-28

**Authors:** Jo-Wei Allison Hsieh, Pei-Yu Lin, Chi-Ting Wang, Yi-Jing Lee, Pearl Chang, Rita Jui-Hsien Lu, Pao-Yang Chen, Chung-Ju Rachel Wang

**Affiliations:** ^1^ Institute of Plant and Microbial Biology, Academia Sinica, Taipei, Taiwan; ^2^ Genome and Systems Biology Degree Program, Academia Sinica and National Taiwan University, Taipei, Taiwan; ^3^ Department of Tropical Agriculture and International Cooperation/Department of Biological Science and Technology, National Pingtung University of Science and Technology, Pingtung, Taiwan

**Keywords:** maize ATAC-seq, chromatin accessibility, chromatin structure, ATAC-seq protocol, next-generation sequencing

## Abstract

The advent of next-generation sequencing in crop improvement offers unprecedented insights into the chromatin landscape closely linked to gene activity governing key traits in plant development and adaptation. Particularly in maize, its dynamic chromatin structure is found to collaborate with massive transcriptional variations across tissues and developmental stages, implying intricate regulatory mechanisms, which highlights the importance of integrating chromatin information into breeding strategies for precise gene controls. The depiction of maize chromatin architecture using Assay for Transposase Accessible Chromatin with high-throughput sequencing (ATAC-seq) provides great opportunities to investigate cis-regulatory elements, which is crucial for crop improvement. In this context, we developed an easy-to-implement ATAC-seq protocol for maize with fewer nuclei and simple equipment. We demonstrate a streamlined ATAC-seq protocol with four key steps for maize in which nuclei purification can be achieved without cell sorting and using only a standard bench-top centrifuge. Our protocol, coupled with the bioinformatic analysis, including validation by read length periodicity, key metrics, and correlation with transcript abundance, provides a precise and efficient assessment of the maize chromatin landscape. Beyond its application to maize, our testing design holds the potential to be applied to other crops or other tissues, especially for those with limited size and amount, establishing a robust foundation for chromatin structure studies in diverse crop species.

## 1 Introduction

In eukaryotes, genomic DNA is packaged with histone proteins, forming nucleosomes that constitute the structural basis of chromatin (Kornberg and Lorch, 1999). The density of nucleosomes determines chromatin compactness and DNA accessibility, which regulate various cellular and chromosomal functions (Hsieh and Fischer, 2005). Genomic regions with dense nucleosomes are tightly packed (i.e., “closed”), whereas nucleosome-depleted regions with exposed DNA are more accessible (i.e., “open”), so the dynamic structures of chromatin provide different levels of availability of DNA binding sites in regulatory regions to transcription factors (Wolffe, 1997). It is now evident that chromatin structure is intimately linked to the activity of underlying genes, thus influencing proper development and the ability to adapt to an ever-changing environment.

Over the last decade, the development of a wide range of methods that utilize nuclease enzymes such MNase and DNase I to target open DNA regions, combined with next-generation sequencing (NGS), has enabled genome-wide investigations of chromatin accessibility (Zhang and Pugh, 2011; Meyer and Liu, 2014). For example, MNase digests the linker DNA between nucleosomes, so subsequent NGS reads largely represent the footprints of nucleosomes, which can be used to assess nucleosome occupancy (Chodavarapu et al., 2010). Another method utilizes an optimized concentration of non-specific endonuclease DNase I that generates DNA fragments by liberating open chromatin regions. The resulting NGS reads are characterized as DNase I hypersensitive sites (DHSs), which represent accessible regions of chromatin (Valouev et al., 2011). These methods have been implemented in a number of organisms, ranging from yeast to plants and human. However, they usually require millions of cells, empirical enzymatic titrations, as well as several purification steps, rendering them challenging for reproducible evaluation of chromatin status and consequently, they are infeasible for some cell types (Song and Crawford, 2010).

An improved method for identifying accessible chromatin is the Assay for Transposase-Accessible Chromatin with high-throughput sequencing (ATAC-seq) (Buenrostro et al., 2013, 2015). This method takes advantage of the engineered prokaryotic Tn5 transposase (Tn5p) that cleaves DNA in open chromatin regions and simultaneously integrates adapters into cleavage sites *in vivo*. Due to the integrated adapters loaded by Tn5p, the ligation and additional purification steps prior to sequencing necessary in other methodologies are eliminated. Using simple library amplification steps, a very small amount of starting material has been successfully used to profile genome-wide chromatin accessibility (Buenrostro et al., 2013). Several studies have shown that 500–50,000 nuclei are sufficient for NGS analyses, considerably less than the nuclei required for other methods (Buenrostro et al., 2013; Gray et al., 2017). Thus, the small amount of cell numbers needed and its high resolution for mapping open chromatin regions make ATAC-seq an excellent tool for genome accessibility profiling.

ATAC-seq has been used to profile the chromatin structure of various animal samples (Corces et al., 2017; Gray et al., 2017; Shashikant and Ettensohn, 2019), but such analyses on plants remain more challenging (Wilkins et al., 2016; Lu et al., 2017; Maher et al., 2018). Unlike animal samples, where cells can be directly used as input for the transposition reaction immediately after lysis with a gentle detergent, plant tissues require physical disruption to release cell contents from the rigid cell walls. Thus, various methods were attempted to break down plant cell walls for plant ATAC-seq, such as grinding samples in liquid nitrogen (Galli et al., 2018; Deschamps et al., 2021; Wu et al., 2023), digesting cell walls for protoplast preparation (Dong et al., 2017; Dai et al., 2022), or disrupting plant tissues using homogenizer or razor blade (Lu et al., 2017; Ricci et al., 2019). In addition to cell walls, another obstacle in plants is the higher quantity of organelles (i.e. mitochondrial and chloroplast) compared to animal cells. As Tn5p targets not only nuclear DNA but also organellar DNA, the presence of a significant amount of organellar DNA can lead to up to 50% of sequencing reads being unusable (Montefiori et al., 2017). To purify plant nuclei from organelles, flow cytometry is commonly used, where nuclei stained with DAPI or expressing fluorescent proteins are sorted (Lu et al., 2017; Galli et al., 2018; Noshay et al., 2019; Ricci et al., 2019; Wu et al., 2023). Alternatively, Deal and Henikoff developed a method, named the isolation of nuclei tagged in specific cell types (INTACT), which involves applying streptavidin-coated magnetic beads to isolate biotin-labeled nuclei extracted from plant samples (Deal and Henikoff, 2011). Although this method allows for the purification of large numbers of nuclei, it requires generating transgenic plants, which could be time-consuming and not practical for many species, especially crops with low transformation efficiency.

Maize (*Zea mays*) is one of the most important crops globally, being widely consumed as staple food and livestock feed, as well as for its industrial uses. The maize genome is relatively large (~2.3 Gbp) and has a complex organization of interspersed genes that are separated by transposable elements (Schnable et al., 2009). In fact, 85% of genes are positioned within 1 kb of transposons nearby (West et al., 2014). How maize cells coordinate this complicated chromatin structure with the transcription program in different tissues and developmental stages is a fascinating biological question. Many studies have explored massive transcriptional variation in different maize tissues and among different maize inbred lines (Dong et al., 2012; Chen et al., 2014; Walley et al., 2016). Interestingly, epigenetic analyses—such as DNA methylation, siRNA profiling, nucleosome occupancy and histone modification profiles—have suggested that epigenetics plays regulatory roles in gene expression and various aspects of maize development (Wang et al., 2009; Gent et al., 2013; Makarevitch et al., 2013; Rodgers-Melnick et al., 2016; Hsu et al., 2017; Oka et al., 2017; Zhao et al., 2018). Its genome complexity and intricate interplay between different levels of epigenetic control make maize an interesting model. Although the maize genome has been sequenced and related epigenetic features are being actively studied, its chromatin accessibility profile remains less investigated.

**Figure 1:**
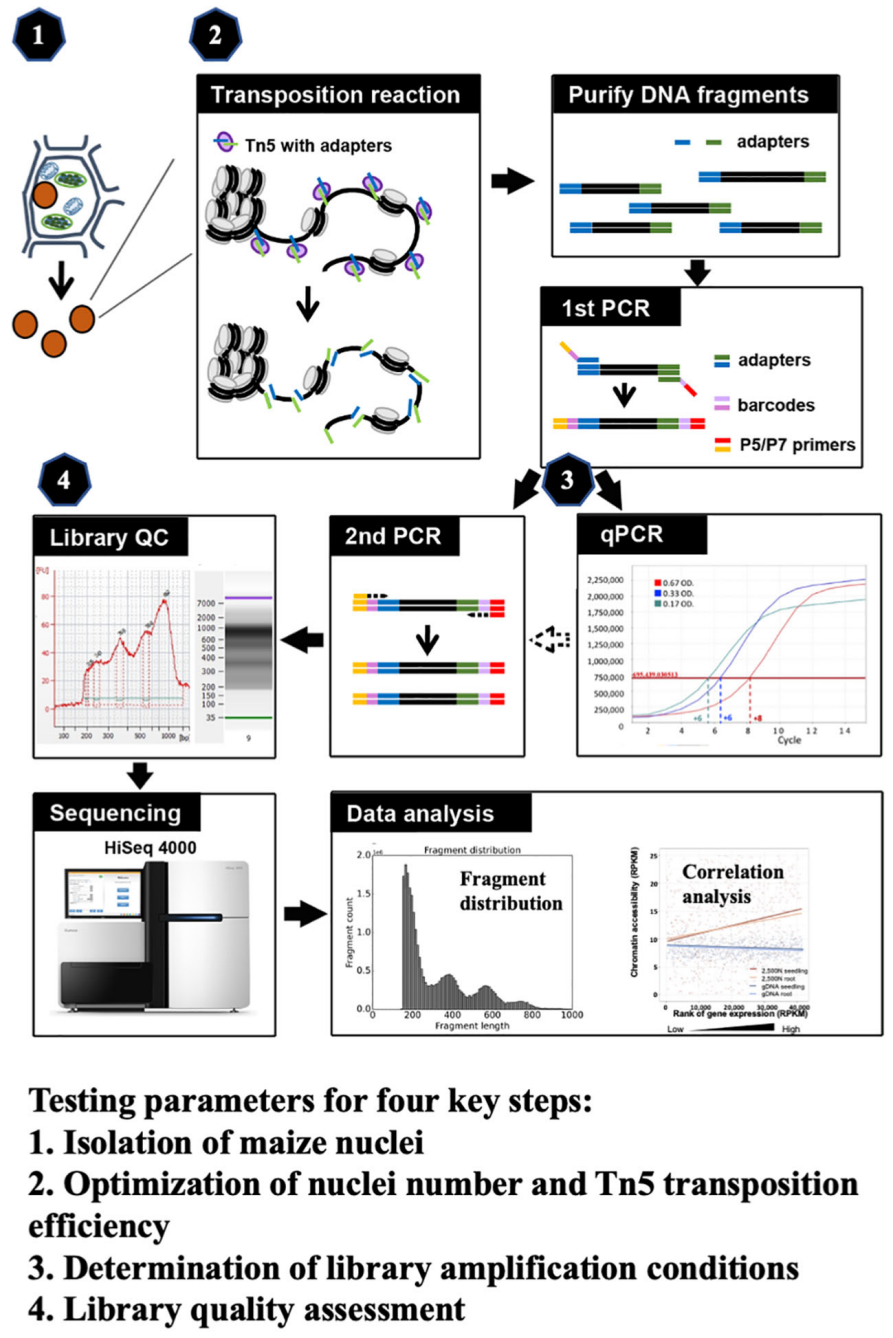
The schematic of maize ATAC-seq protocol, highlighting four key steps that were optimized: (1) isolation of maize nuclei with reduced organelle contamination; (2) optimization of nuclei number and Tn5p transposition efficiency to mitigate over-reaction and increase effectiveness; (3) determination of library amplification conditions to minimize excessive PCR duplications; and (4) library quality assessment prior to the costly and time-consuming sequencing process.

ATAC-seq has been employed in maize genome research, where most ATAC-seq results derive from tens of thousands of nuclei isolated by flow cytometry or prepared from mesophyll protoplasts (Dong et al., 2017; Galli et al., 2018; Noshay et al., 2019; Ricci et al., 2019; Crisp et al., 2020; Wu et al., 2023). To make the ATAC-seq method more applicable easily, we optimize critical steps in this study and develop a robust and efficient methodology for ATAC-seq analysis in maize. We meticulously evaluated four key components (as illustrated in **Figure 1**), including (1) the isolation of maize nuclei; (2) the optimization of nuclei number and Tn5p transposition efficiency; (3) determination of library amplification conditions; and (4) the assessment of library quality. All tested parameters and their corresponding results are presented, and the optimized protocol is described in the step-by-step method details. Finally, we validated our ATAC-seq results by examining fragment size distribution, key metrics, and the correlation with RNA-seq data. Through optimization of critical steps and quality assessments, this refined protocol provides an efficient assessment of the maize chromatin landscape and can be adapted for other plant species.

## 2 Materials and equipment

### 2.1 Biological materials

- 10-day-old maize B73 seedlings

### 2.2 Reagents

#### 2.2.1 Chemicals and enzymes

- Percoll (Sigma-Aldrich, Cat#P7828)
- 3-(N-morpholino) propanesulfonic acid, 4-morpholinepropanesulfonic acid (MOPS) (Merck, Cas#1132612)
- 10N NaOH (Merck, Cat# 1310732)
- 5M NaCl (Merck, Cat#7647145)
- 3M KCl (Merck, Cat# 7447407)
- 500 mM EDTA (Invitrogen, Cat#15575020)
- 100 mM EGTA (Sigma-Aldrich, Cas#13368133)
- 400 mM spermine (Sigma-Aldrich, Cat#85590)
- 400 mM spermidine (Sigma-Aldrich, Cas#124209)
- cOmplete™, EDTA-free Protease Inhibitor Cocktail (Roche, Cat#04693159001)
- Sucrose (Sigma-Aldrich, Cas#57501)
- Tris base (Sigma-Aldrich, Cat#77861)
- 100 mM MgCl2 (Invitrogen, Cat#AM9530G)
- Triton X-100 (Sigma-Aldrich, Cat#9036195)
- 4’,6-diamidino-2-phenylindole (DAPI)
- Ethanol (Merck, Cat#1.00983.2500)
- UltraPure DNase/RNase-Free Distilled Water (Thermo Fisher Scientific, Cat#10977015)
- Buffer EB (Qiagen, Part#19086)

#### 2.2.2 Critical commercial assays

- Illumina Tagment DNA TDE1 Enzyme and Buffer Kits (Illumina, Cat#20034197)
- Qubit dsDNA BR Assay Kit (Thermo Fisher Scientific, Cat#Q32853)
- NEBNext High-Fidelity 2x PCR Master Mix (New England Labs, Cat#M0541S)
- Nextera Index Kit (Illumina, Cat#FC-121–1011)
- MinElute PCR Purification Kit (Qiagen, Cat#28006)
- AMPure beads (Beckman, Cat#A63880)
- KAPA SYBR^®^ FAST qPCR Master Mix (Roche, Cat#07960204001)
- Qubit dsDNA High Sensitivity Assay Kit (Thermo Fisher Scientific, Cat#Q32851)
- High Sensitivity DNA Kit (Agilent Technologies, Part#5067–4626)

### 2.3 Solutions

*1 ml of Protease inhibitor solution (50x)*

Mix by vertexing and store at -20°C for up to 12 weeks.

- complete™, EDTA-free protease inhibitor cocktail tablet - 1 tablet
- Milli-Q water - 1 ml

*100 ml of 20% (v/v) Triton X-100*

Stir for 30 minutes until fully mixed. Store in the dark at room temperature for a maximum of 2 months.

- Triton X-100 - 20 ml
- Milli-Q water - 80 ml

*10 ml of Organelle Removal Buffer (ORB)*

Store at 4°C for 1 week. Add the protease inhibitor stock solution right before use.

- 2.5 M Sucrose stock solution - 1 ml (250 mM)
- 1M, PH8.0 Tris-HCl - 100 μl (10 mM)
- 100 mM MgCl_2_ - 1 ml (10 mM)
- 20% Triton X-100 - 500 μl (1%)
- 50x Protease inhibitor stock solution - 200 μl
- Milli-Q water - 7.2 ml

*10 ml of Sucrose Cushion Buffer (SCB)*

Store at 4°C for 1 week. Add the protease inhibitor stock solution right before use.

- 2.5 M Sucrose stock solution - 6.8 ml (1.7 M)
- 1M, PH8.0 Tris-HCl - 100 μl (10 mM)
- 100 mM MgCl_2_ - 200 μl (2 mM)
- 20% Triton X-100 - 50 μl (0.1%)
- 50x Protease inhibitor stock solution - 200 μl
- Milli-Q water - 2.65 ml

*100 ml of MOPS (1M)*

Autoclave and store at 4°C for a maximum of 3 months.

- 3-(N-morpholino) propanesulfonic acid, 4-morpholinepropanesulfonic acid (MOPS) - 20.93 g (1 M)
- Milli-Q water - 80 ml
- NaOH (10N) - Adjust to pH7.0

*10 ml of Nucleus Extraction Buffer (NEB)*

Store at 4°C for 1 week. Add protease inhibitor, spermine, and spermidine stock solutions right before use.

- 1 M, pH 7.0 MOPS - 200 μl (20 mM)
- 5 M NaCl -80 μl (40 mM)
- 3 M KCl - 300 μl (90 mM)
- 500 mM EDTA - 50 μl (2.5 mM)
- 100 mM EGTA - 50 μl (0.5 mM)
- 400 mM Spermine - 5 μl (0.2 mM)
- 400 mM Spermidine - 13 μl (0.52 mM)
- 50x Protease inhibitor solution - 200 μl
- Milli-Q water - 9.1 ml

*DAPI stock solution*

Dissolve DAPI with vigorous shaking. To prepare the working DAPI solution, dilute the stock solution 1000 x to 1 µg/ml. For long-term storage, aliquot the stock solution and store at -20°C, where it will remain stable for at least six months.

- DAPI dilactate - 10 mg
- Milli-Q water - 1 ml

### 2.4 Equipment and materials

- Long gel-loading pipette tips (Labcon, Part#1034–960-008)
- Double-edged stainless steel razor blade (Electron Microscopy Sciences)
- Glass Petri dish
- Parafilm
- MiraCloth
- CellTrics cell strainers (10 and 20 µm mesh) (Sysmex Partec)
- Hemocytometer
- 1-ml and 2-ml Eppendorf low-binding tubes
- 1.7ml Microtube, Clear, Maxymum Recovery (Axygen, Part#MCT-175-L-C)
- Fine nylon paintbrush (size 0)
- Thermomixer Comfort (Eppendorf, Cat#5355)
- T100™ Thermal Cycler (BIO-RAD, Cat#186–1096)
- Centrifuge: MiniSpin Plus (Eppendorf, Cat#5453)
- Applied Biosystems QuantStudio™ 12K Flex Real-Time PCR System (Thermo Fisher Scientific, Cat#4470050)
- DYNAL MPC-S magnetic stand (Applied Biosystems)
- Qubit™ 2.0 (Thermo Fisher Scientific, Cat#Q32866)
- Agilent 2100 Bioanalyzer Instrument (Agilent Technologies, Cat#G2939BA)

### 2.5 Software and algorithms

- R version 3.4.4 (https://www.r-project.org)
- FastQC v0.11.8 (http://www.bioinformatics.babraham.ac.uk/projects/fastqc/)
- TrimGalore v0.4.1 (http://www.bioinformatics.babraham.ac.uk/projects/trim_galore/)
- HISAT2 v2.1.0 (http://daehwankimlab.github.io/hisat2/)
- edgeR v3.20.9 (https://bioconductor.org/packages/release/bioc/html/edgeR.html)
- Bowtie2 v2.3.5.1 (http://bowtie-bio.sourceforge.net/bowtie2/)
- ATACgraph (https://github.com/RitataLU/ATACgraph)

## 3 Methods

### 3.1 Step-by-step method details

#### 3.1.1 Nuclei isolation

A crucial requirement for generating a good ATAC-seq library is to use pure and intact nuclei (Lu et al., 2017; Marand et al., 2021). Notably, since Tn5p targets not only nuclear DNA but also organellar DNA, the sequencing reads from organellar DNA fragments can often account for up to 50% of the total sequencing reads (Montefiori et al., 2017). Therefore, the removal of organelles prior to the transposition reaction can thus greatly improve ATAC-seq efficiency. In the process of isolating plant nuclei, two steps are typically involved: breaking plant cell walls to release nuclei, and removing the organelles.

##### Timing 1–2 h

1. Cool the centrifuge to 4°C. Keep reagents and solutions on ice throughout the process.

2. Prepare the Percoll-Sucrose gradient buffer. Mix 400 µl Organelle Removal Buffer (ORB) with 600 µl Percoll to make 60% Percoll solution. Add 200 µl Sucrose Cushion Buffer (SCB) to a new 1.5-ml microcentrifuge tube. Carefully overlay with 400 µl 60% Percoll solution. Be very careful not to mix the sucrose and Percoll layers. Keep the gradient on ice. Perform this step at least 1 hour before using the Percoll-Sucrose gradient.

**Note:** We found that one-hour equilibration of the Percoll-Sucrose gradient buffer resulted in better nuclei recovery than immediate use, as incorrect Percoll concentration may affect gradient formation and stability, leading to irregular sample sedimentation.

**Figure 2:**
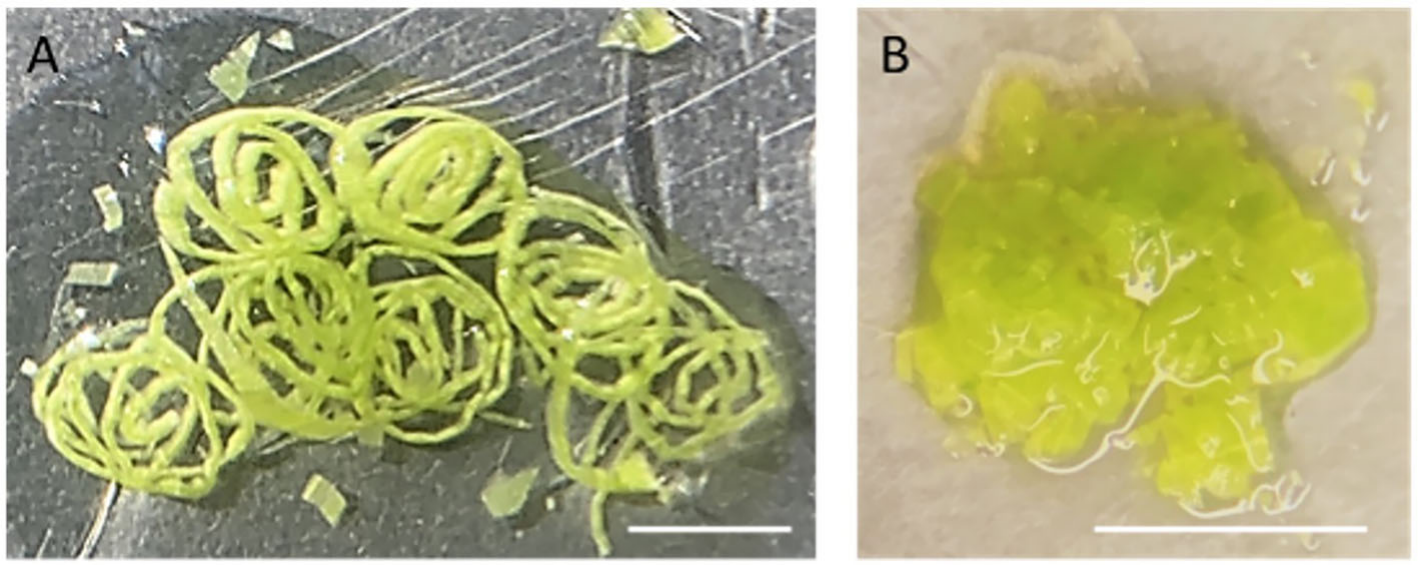
Chopped leaf tissue on a piece of parafilm. **(A)** The rolled leaves were first sliced into thin strips. **(B)** After fine chopping, the leaf tissue has the appearance of a coarse mixture. Scaled bar represents 5mm.

**Note**: Chopping with a sharp razor blade is a gentler method to release nuclei, minimizing potential damage compared to grinding tissues directly in liquid nitrogen. To assist free-hand chopping, leaves were first rolled up and sliced finely across the veins into thin strips. Subsequently, these strips are then chopped into a coarse mixture.

3. Place a piece of 5x5 cm^2^ parafilm on a glass Petri dish on ice. Pipette 500 µl NEB onto the parafilm and finely slice the above-ground parts of 10-day-old maize seedlings (approximately 5 seedlings) in the NEB buffer with a double-edged razor blade. After slicing, proceed to chop further in 2–3 separate batches until the tissue appears like a coarse mixture (**Figure 2**). Each batch of chopping takes about 2–5 minutes to complete. Add more NEB buffer if needed. Approximately 5 ml NEB is required for 5 seedlings.

4. Transfer the mixture to a small petri dish on ice. Incubate for 5 minutes with gentle agitation. Place 4 layers of Miracloth on a pre-chilled glass funnel. Filter the mixture through the funnel into a 15-ml Falcon tube. Gently squeeze the remaining tissue against the funnel to extract more nuclei.

5. Filter the crude nuclei suspension through a 20-µm CellTrics twice to remove large debris. Divide the filtrate into 1.5-ml microcentrifuge tubes and centrifuge at 800 rcf for 10 minutes at 4°C.

6. Gently remove and discard the supernatant without disturbing the pellet. The pellet should comprise a pale-white layer at the bottom and a green layer on top.

**Note**: The pale-white layer predominantly consists of starch grains, and the top layer contains nuclei, chloroplasts and other organelles.

7. Slowly pipette 200 µl ORB (with 1% Triton X-100) into each tube from step 6 and incubate for 2 minutes so the pellet loosens. Use a fine nylon paintbrush to gently remove the green layer from the pellet, leaving the pale-white bottom layer undisturbed. Gently transfer the green pellets with ORB to a new tube by pipetting. Resuspend the green pellet material in the ORB using the paintbrush and by gentle pipetting.

**Note**: Chloroplasts are lysed in 1% Triton X-100 in the ORB buffer. If this leftover solution is centrifuged, the pellet will be white and lack the uppermost green layer as chlorophyll is released in the supernatant from broken chloroplasts.

8. Filter the nuclei suspension from step 7 through a 10-µm CellTrics twice to remove aggregate nuclei and debris.

9. Carefully load approximately 400 µl of the filtered green-colored nuclei suspension on top of the previously prepared Percoll-Sucrose gradient buffer. Centrifuge at 1,000 rcf for 15 minutes at 4°C. Prepare an additional Percoll-Sucrose gradient, if more than 400 µl nuclei suspension solution is being used.

10. Remove the green-colored supernatant on the top. Collect the second layer of 60% Percoll buffer that contains intact nuclei and transfer the nuclear fraction into a new 1.5-ml microcentrifuge tube. Be careful not to disturb the interface between Percoll and sucrose layers.

**Note:** As we have not tested this protocol in other organisms, users may need to verify the presence of intact nuclei under a microscope when applying it to different species. Although untested, the flowchart and concept of our protocol could serve as a reference to help scientists conduct ATAC-seq in other organisms.

11. Add 1X volume of NEB to dilute the nuclear fraction suspension. Centrifuge at 500 rcf for approximately 10 minutes to pellet the nuclei.

**Note**: Check the pellet condition every 2 minutes and stop centrifugation when a small translucent pellet is first visible. We found that excessive centrifugation can result in nuclear damage. The nuclear pellet is translucent, different from the pale white color of the starch grain pellet from step 6.

**Figure 3:**
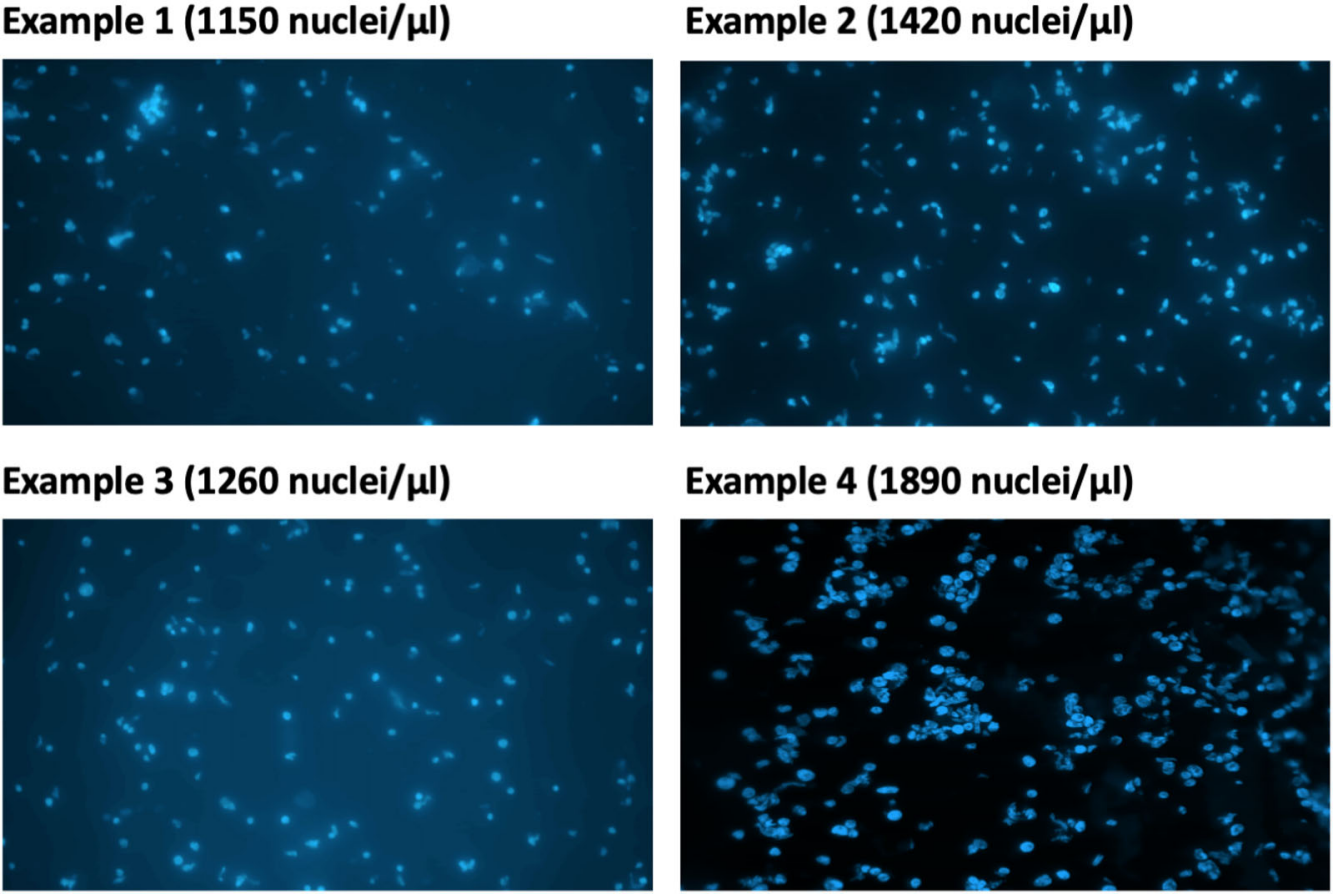
Estimation of DAPI-stained nuclei under microscope.

**Note**: The expected concentration of nuclei in the isolated nuclei solution from step 12 is approximately 5–20 million/ml.

12. Dissolve the pellet in 20 µl NEB to obtain the final isolated nuclei solution.

13. Take 2 µl of the isolated nuclei solution from step 12 and dilute in NEB. Stain with DAPI (final concentration: 0.3 µg/ml) (**Figure 3**). Examine the nuclei integrity under a microscope and calculate nuclei density of the isolated nuclei suspension using a hemocytometer.

14. Transfer a volume equivalent to 2,500 or 5,000 nuclei to a new 1.5-ml low-binding tube.

**Note**: According to the manufacturer’s manual of Nextera DNA Library Preparation Kit, the input volume of nuclei for the transposition reaction should be < 5 µl.

#### 3.1.2 Tn5p Transposition

The Nextera DNA Library Preparation Kit (FC-121–1031, Illumina) was used to perform the transposition reaction according to the manufacturer’s manual. In addition to nuclear samples, 50 ng genomic DNA (gDNA) was used as a negative control input. A brief description of the procedure is as follows.

##### Timing 40 minutes

15. Prepare the transposition reaction mix and gently resuspend the nucleus pellet in the transposition reaction mix as follows.

**Note**: For maize, we found that only about 2,500 and 5,000 nuclei are good starting materials are required for a single reaction. As we have not tested this protocol in other organisms, when adapting this protocol for different species, it is recommended to optimize the number of nuclei used in one reaction.

- Purified intact nuclei (2,500 or 5000 nuclei) or 50 ng gDNA - 2 μl
- 2 x TD reaction buffer - 25 μl
- TDE1 (Nextera Tn5 Transposase) - 2.5 μl
- Nuclease free water - 20.5 μl

16. Incubate the transposition reaction at 37°C for 30 minutes in an Eppendorf ThermoMixer Comfort with agitation at 2 rcf for 2 minutes, followed by a rest period of 2 minutes (i.e., occasionally mixing).

**Note**: Occasional gentle mixing may facilitate uniform reaction and increase fragment yield.

17. Immediately following transposition, purify the Tn5p transposed DNA fragments with a Qiagen MinElute PCR Purification Kit.

#### 3.1.3 Purification of DNA fragments

Use a Qiagen MinElute PCR Purification kit to purify the transposed DNA fragments according to the manufacturer’s manual. A brief description of the procedure is as follows.

##### Timing 15 minutes

18. Add 5X volume of PB Buffer into the transposition reaction and vortex to mix.

**Note**: Check for the yellow color of PB Buffer. If the buffer turns orange or violet, the pH is too high. A small volume of 3M sodium acetate can be added to adjust the pH before proceeding.

19. Transfer the mix into a MinElute spin column provided by the Qiagen MinElute PCR Purification kit.

20. Centrifuge for 1 minute at 16,000 rcf. Discard all the flow-through.

21. Add 750 μl of PE Buffer, and centrifuge for 1 minute at 16,000 rcf. Discard the flow-through.

22. Centrifuge for 1 minute at 16,000 rcf. Rotate the column by 180°C and centrifuge for 1 minute.

**Note**: This step removes any remaining ethanol in the column.

23. Place the MinElute spin column in a new low-binding tube.

24. Elute in a new tube with 12 μl of EB buffer (10 mM Tris, pH 8.5) and stand at room temperature for 1 minute.

25. Centrifuging for 1 minute at 16,000 rcf. Rotate the column 180°C and centrifuge for 1 minute.

26. Discard the column and store the purified DNA at -20°C if necessary.

**Note**: This is a convenient stopping point for the protocol.

#### 3.1.4 Preparation of sequencing libraries

This phase consists of four major steps. First, indexing barcodes are added onto each transposed DNA fragment by 5 cycles of PCR amplification (‘the first PCR’). Second, a small aliquot from the product of the first PCR is subjected to quantitative PCR (qPCR) to assess the amount of DNA template, and the result will be used to determine the number of cycles used in the secondary PCR. Third, these indexed fragments from the first PCR are further amplified with sequencing primers (i.e., ‘the secondary PCR’) to generate a sufficient amount of DNA for sequencing. Fourth, the resulting library from the secondary PCR is purified.

##### Timing 3–4 hours

27. First PCR amplification with indexing barcode primers.

Use the Nextera Index Kit and NEBNext High-Fidelity 2x PCR Master Mix to amplify transposed DNA fragments. A brief description of the procedure is as follows.

- Prepare a PCR mixture by combining the First PCR Amplification Reagent with 10 µl purified transposition product in a 0.2 ml PCR tube as follows.

**Note**: Different index primers (barcodes) should be selected for distinct samples, so that these samples can be pooled together for sequencing. After sequencing, reads from these samples can be identified and separated according to their barcodes.

• Transposed DNA - 10 μl
• NEBNext High-Fidelity 2x PCR Master Mix - 25 μl
• 25 μM Index 1 (i7) - 2.5 μl
• 25 μM Index 2 (i5) - 2.5 μl
• Nuclease free water - 10 μl

b. The thermal cycle for PCR amplification is as follows: 72°C for 5 minutes, 98°C for 30 seconds, 5 cycles (98°C for 10 seconds, 63°C for 30 seconds, and 72°C for 1 minute).
c. Transfer the PCR product to a new microcentrifuge tube.

28. Determination of cycle number for the secondary PCR by qPCR.

The KAPA Library Quantification Kit is used for the quantitative PCR. A brief description of the procedure is as follows.

- Take 1 μl of the amplification product from the first PCR reaction and mix with qPCR reagents as follows.
• first PCR product amplification product - 1 μl
• 2X KAPA SYBR^®^ FAST qPCR Master Mix with 10X • Primer Premix -12 μl
• Nuclease free water - 7 μl
- Perform the qPCR amplification on an Applied Biosystems QuantStudioTM 12K Flex Real-Time PCR System with the following conditions: 98°C for 5 minutes, 20 cycles (98°C for 10 seconds, 63°C for 30 seconds, and 72°C for 1 minute).
- Calculate the required number of cycles (i.e., ‘N’ cycles) for the secondary PCR step by plotting linear Rn versus the cycle number. The N cycle number corresponds to the cycle number on the qPCR plot, where the fluorescent intensity reaches one-third of the maximum value.

29. Secondary PCR amplification.

Conduct the secondary PCR using the remaining product from the first PCR as the template, mixed with the NEBNext High-Fidelity 2x PCR Master.

- Prepare the Secondary PCR Amplification reagents and add 40 μl of the first PCR product from step 27 in a 0.2 ml PCR tube as follows.
• first PCR product amplification product - 40 μl
• NEBNext High-Fidelity 2x PCR Master Mix - 50 μl
• PCR Primer Cocktail - 5 μl
• Nuclease free water - 5 μl
- Perform the secondary PCR amplification as follows: 98°C for 30 seconds, and N cycles of (98°C for 10 seconds, 63°C for 30 seconds, and 72°C for 1 minute). The N cycle number is determined based on the qPCR analysis in Step 28.

**Note**: While the original protocol suggests using 5 µl of the first PCR product as the template for qPCR, we have found that 1 µl is sufficient (step 28). Consequently, approximately one-fifth of the DNA template is used to estimate the “N” cycle number in our assay, which equates roughly to two cycles of PCR amplification. Therefore, in our experience, successful libraries can be generated using a cycle number lower than what is determined by qPCR (step 28). This adjustment helps in avoiding over-amplification.

30. Purification of the amplified library.

Use the AMPure XP kit to purify the amplified DNA from step 29. A brief description of the procedure is as follows.

- Allow the AMPure XP beads to reach room temperature for at least 30 minutes. Vortex the AMPure XP Beads until they are well dispersed. Add 100 μl (1X volume) of well-mixed AMPure XP Beads into a new 1.7 ml tube.
- Transfer each DNA library from step 29 to the 1.7 ml tube containing the AMPure XP Beads. Gently pipette the entire volume up and down 10 times or vortex gently to mix thoroughly.
- Incubate the tubes at room temperature for 5 minutes. Place the tubes on a magnetic stand at room temperature for 3 minutes or until the liquid appears clear. Carefully remove the supernatant from each tube.

**Note**: Some liquid may remain in each tube. Do not disturb the beads.

d. Wash the bead-bound DNA pellet twice with 200 μl of 80% ethanol. Let the tubes open at room temperature for 10 minutes, enabling the pellet to dry.
e. Resuspend the dried pellet in each tube with 22.5 μl EB Buffer. Vortex to mix thoroughly and incubate the tube at room temperature for 2 minutes.
f. Place the tube on a magnetic stand at room temperature for 3 minutes or until the liquid appears clear. Transfer all of the clear supernatant, which contains the purified DNA, to a new 1.7 ml tube. The samples can be stored at -20°C.

#### 3.1.5 Checking library quality and sequencing

31. Use the Qubit High Sensitivity Assay Kit to determine the concentration of each ATAC-seq library.

32. Assess the fragment size distribution and peak pattern of each library using either the Agilent Bioanalyzer 2100 system or BiOptic Qsep400.

**Note**: A successful ATAC-seq library should exhibit a pattern of periodicity in fragment sizes with an interval of around 200 bp.

33. We used an Illumina platform HiSeq 4000 for 150-bp paired-end sequencing to generate 30 million raw reads for each maize library.

**Note**: Other Illumina sequencing platforms (e.g., Hiseq X Ten, and NovaSeq 6000) can be used. Choose ones capable of paired-end sequencing with at least read length of 50 bp.

### 3.2 Quantification and statistical analysis pipeline

#### 3.2.1 RNA-seq analysis pipeline

The RNA-seq reads derived from maize seedling and root samples (accession numbers SRR7548392 and SRR2043190) are used for analysis. The bioinformatics pipeline for the basic analysis of the RNA-seq dataset is outlined as follows, including quality control to the calculation of Fragments Per Kilobase Million (FPKM).

1. Each sample is quality checked by FASTQC v0.11.8.
2. The adapter and quality were trimmed using TrimGalore v0.4.1 (Krueger et al., 2016), and at least 35 bp long reads were retained, obtaining clean FASTQ files.
3. Reads were aligned to the Maize B73 reference genome (AGPv4) using HISAT2 v2.1.0 (Kim et al., 2019) (**Supplementary Table 1**).
4. Read counts were normalized using the TMM function in edgeR package v3.20.9 (Chen et al., 2016) under R v3.4.4, and then FPKM values were obtained by calling function of rpkm. The ranks of transcript abundance are listed by its log_2_ FPKM from the lowest to the highest for its correlation analysis with chromatin accessibility.

#### 3.2.2 ATAC-seq analysis pipeline

The pipeline for the ATAC-seq data analysis is adopted from ATACgraph (Lu et al., 2020). The code used here is available at GitHub: https://github.com/beritlin/ATACgraph2 (Lin, 2023). It is specifically designed for ATAC-seq data analysis and is able to remove mitochondria and plastid DNA, identify open regions by peak calling, plot the fragment length distribution, compute the periodicity of fragment length distribution using FFT algorithms as well as show heatmaps showing accessibility around all genes.

1. The raw ATAC-seq reads were underwent trimming, duplication removal, and aligned to the maize reference genome (AGPv4) using Bowtie2 v2.3.5.1 (Langmead and Salzberg, 2012) (**Supplementary Table 2**).
2. Following the steps in ATACgraph, mitochondria and plastid DNA were removed by command line 00_rmChr with Mt and Pt as the option, an aligned bam file as input and obtained clean bam files as output.
3. The clean bam file and its matched gDNA bam file were then used for peak calling. Running the function 03_callPeak with the provided gene body bed file, will generate the peak location narrowPeak files, intensity bigwig (bw) files, as well as a genes list of overlapping with peak locations txt file as the selected output name in the command. The script is updated to MACS3 and the parameter here is using p-value < 0.05 as a cutoff for peaks calling due to the small amount of nuclei used in our protocol.
4. Figures were also plotted by ATACgraph, including the period of fragment length distribution and FFT by implementing 01_calFragDist by using clean bam file as input. Heatmaps around genes was regenerated by command line 03_genePlot with narrowPeak file, bigwig (bw) and the bed files.
5. The read counts at the peak regions were generated by computeMatrix v3.3.2 from the bigwig (bw) file and normalized using the TMM function in edgeR package v3.20.9 under R v3.4.4.
6. The evaluation of libraries was also performed by running the following commands, providing the peaks files, bam files, and gene annotation which returns the log file of each score.

> ATACgraph 00_rmChr atac_sample.bam atac_sample_rmM.bam Mt, Pt

> ATACgraph 03_callPeak atac_sample_rmM.bam atac_sample_rmM_peakcall Maize_gene_body_bed6.bed -c gDNA.bam

> ATACgraph 01_calFragDist atac_sample_rmM.bamatac_sample_fragment atac_sample_FFT

> ATACgraph 03_genePlot atac_sample_rmM_peakcall.narrowpeak atac_sample_rmM_peakcall_coverage.bw Maize

> ATACgraph 04_IDR.py atac_sample_rmM_R1_peakcall.narrowpeak atac_sample_rmM_R2_peakcall.narrowpeak atac_sample_idr

> ATACgraph 04_frip.py atac_sample_rmM_peakcall.narrowpeak atac_sample_frip atac_sample_rmM.bam

> ATACgraph 04_tssEnrich.py atac_sample_rmM_peakcall.narrowpeak Maize atac_sample_tss atac_sample_rmM.bam

## 4 Results

Expected outcomes at each critical step of the protocol are described. Additionally, comparisons of various parameters were carried out to ensure optimized parameters and the effectiveness of ATAC-seq.

### 4.1 Pure and intact nuclei isolation

**Figure 4:**
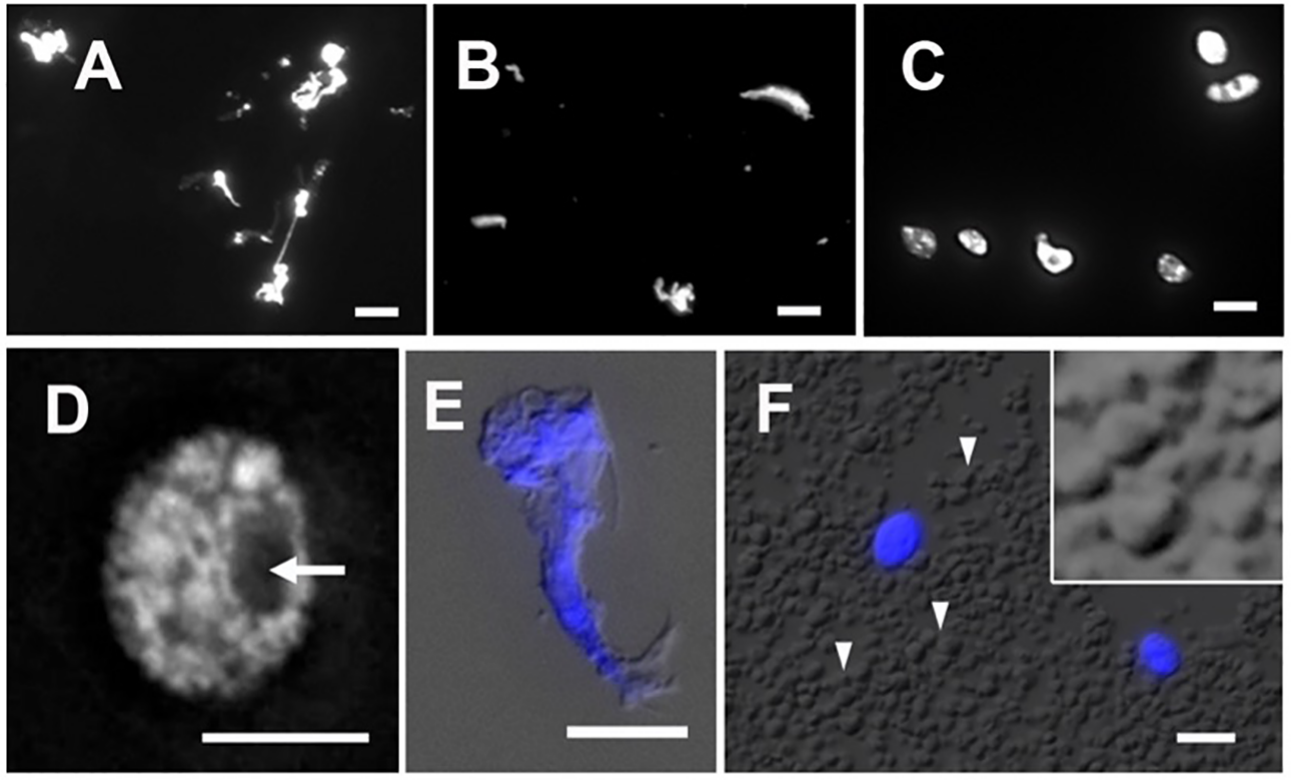
Nuclei stained with DAPI after extraction by **(A)** grinding in liquid nitrogen, **(B)** polytron homogenizer and, **(C)** manual chopping. **(D)** A magnified image of a single nucleus isolated by chopping showing a round-shaped nucleus with distinct chromatin morphology and a visible nucleolus (arrow). **(E)** An example of cell debris demonstrates distorted cell structures and scrambled chromatin with DAPI stain (blue), shown with the merged DIC image. **(F)** In the crude homogenate, a large amount of starch grains (arrowheads) can be observed by the DIC microscope. Note that DAPI-stained round-shape nuclei (blue) are scattered. The magnified inset shows polyhedral starch grains. Scale bars represent 5 μm.

The isolation of plant nuclei typically involves the mechanical disruption of cell walls by grinding in liquid nitrogen or breaking tissues using polytrons or razor blades. The objective of this step is to break down the cell wall and release cellular contents, including nuclei. Since the integrity of nuclei is essential for obtaining qualified data of ATAC-seq, the method ought to be gentle enough to preserve nuclei and chromatin conformation. We found that chopping with a sharp razor blade is a gentler method to release nuclei, minimizing potential damage. To compare nucleus morphology released by different methods, we stained nuclei with DAPI and observed them under a microscope. As shown in **Figures 4A** and **B**, the nuclei extracted by grinding in liquid nitrogen or disrupting by a polytron homogenizer were of poor quality. The stringy mess or misconfigured nuclei suggested that cellular structures are destroyed, and nuclei are damaged. Chromatin released from damaged nuclei exhibited irregular DAPI staining, often seen as scrambled mess with cell debris (**Figure 4E**), so the native chromatin structure likely has been altered. We found that it was difficult to conduct either of these two methods without damaging maize nuclei. In our adapted protocol, where maize fresh tissues are sliced and chopped with a sharp stainless steel razor blade, we successfully obtained high yields of round-shaped nuclei with distinct chromatin morphology (**Figures 4C, D**), in contrast to the damaged nuclei isolated with two other methods (**Figures 4A, B, E**). Interestingly, after collecting nuclei by centrifugation, we found that a large amount of starch grains appeared in a pale white layer underneath a green layer. Starch grains do not stain with DAPI nor show autofluorescence (**Figure 4F**). To minimize starch grains in our samples, we found that a fine nylon paintbrush is very useful to separate the green layer hosting nuclei and most organelles from the underlying starch grain layer (step 7 in the 3.1 section).

To purify nuclei effectively, it is crucial to consider factors such as purity, the required duration, efficiency, and ease of access to equipment. Flow cytometry offers an effective method for isolating intact nuclei from organelles and broken nuclei as it utilizes fluorescent signals (i.e. DAPI-stained nuclei or nucleus-expressed fluorescence) to distinguish nuclei based on their DNA content and size. It is most suitable for collecting hundreds of thousands of nuclei from various debris and organelles. As we wanted to develop a reliable and easy-to-implement ATAC-seq protocol, we optimized the use of the sucrose-Percoll centrifugation method in combination with Triton X-100 treatment, which requires only a bench-top centrifuge.

**Figure 5:**
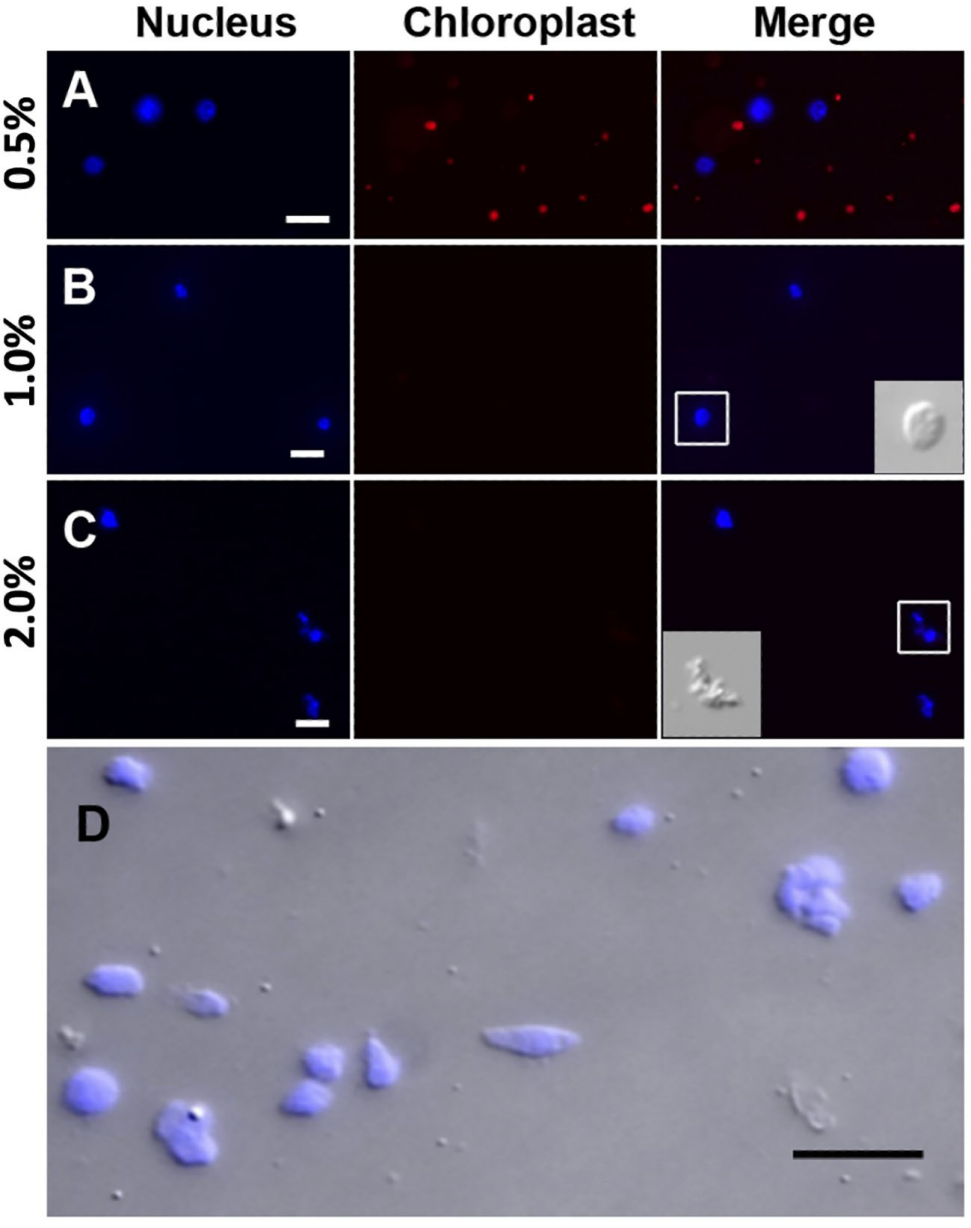
Nuclei treated with different Triton X-100 concentrations: **(A)** 0.5%, **(B)** 1.0%, **(C)** 2.0% Triton X-100 and stained with DAPI (blue). Chloroplasts exhibit strong red autofluorescence. Note that a majority of chloroplasts are lysed in Triton X-100 above 1.0%. When treated with 2.0% Triton X-100, nuclei integrity is severely compromised, as shown in the inset in C by DIC. **(D)** After the sucrose-Percoll gradient centrifugation, most nuclei remain intact. Scale bars represent 10 μm. DAPI-stained nuclei were detected using excitation wavelength 353 nm and emission wavelength 465 nm. Chloroplast autofluorescence was captured using 638 nm/646 nm (excitation/emission).

Prior to the sucrose-Percoll centrifugation (step 9 in the 3.1 section), the non-ionic detergent, Triton X-100, is used to eliminate organelles. We tested different concentrations (0.5–2%) of Triton X-100 to lyze chloroplasts while nuclei remained intact. **Figure 5** shows that both chloroplasts and nuclei remained intact after 0.5% Triton X-100 treatment, but most chloroplasts were lysed in 1% Triton X-100 buffer. However, at higher concentrations of Triton X-100, there was a noticeable decrease of intact nuclei, suggesting that an optimal concentration for lysing chloroplasts, the most abundant organelles, without compromising nuclear integrity, is 1% Triton X-100.

**Figure 6:**
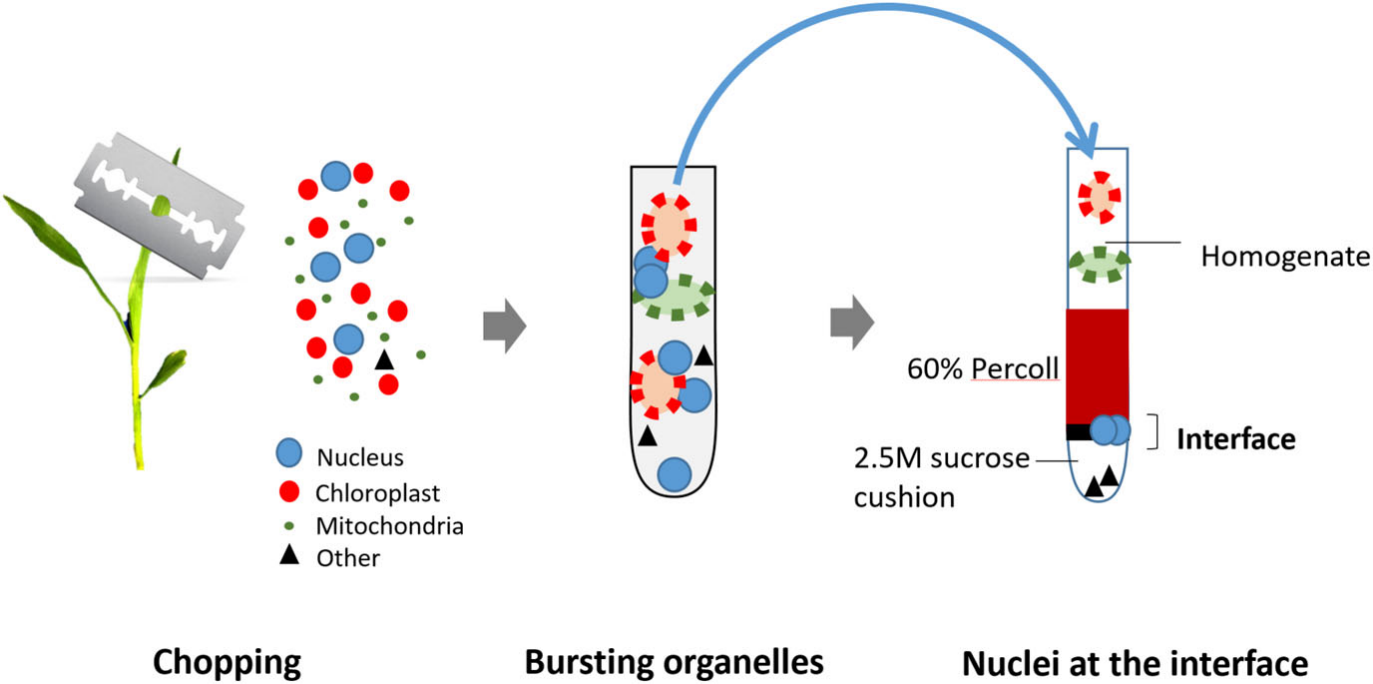
Illustration of the nuclei isolation method from fresh seedlings. The seedlings are first manually chopped in a buffer using a sharp blade to release nuclei and organelles. Next, organelles are lysed using 1% Triton X-100, but nuclei remain intact. The crude exacts are then loaded on the top of Percoll-Sucrose gradient. After centrifugation, maize nuclei are enriched in the 60% Percoll layer. Isolated nuclei are transferred to a new tube and collected by centrifugation for the next transposition reaction. are transferred to a new tube and collected by centrifugation for the next transposition reaction.

**Figure 7:**
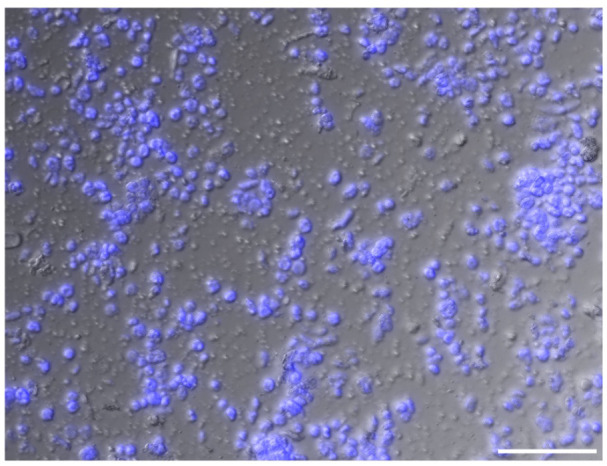
An image of isolated nuclei in high density. Intact nuclei account for approximately 90% of the population. Nuclei are visualized with DAPI stain (blue). Unstained matters include cell debris, a small number of chloroplast, and starch grains. Scale bar represents 50 μm.

The sucrose-Percoll centrifugation method takes advantage of differential buoyant density, size, and shape of subjects, which allows separation of subcellular compartments during centrifugation in high-viscosity media such as sucrose and/or Percoll (a colloidal silica). We tested 60% Percoll with 2.5 M sucrose as a cushion in a bench-top centrifuge (Sikorskaite et al., 2013) (**Figure 6**). After centrifuging at 1,000 rcf for 15 minutes, we observed that the upper layer of supernatant is colored green due to chlorophyll content, and nuclei are suspended in the 60% Percoll layer. A brownish-white layer is sometimes deposited at the sucrose-Percoll interface, which contains mostly contaminating material and intact cells. Nuclei isolated from the Percoll layer were intact with clear nucleoli in microscopic examination. Although some nuclei appeared elongated after centrifugation, their chromatin was well-contained within the nuclear boundary (**Figure 5D**). Taken together, we were able to collect approximately one hundred thousand pure nuclei from five seedlings using this method, which includes manual chopping, 1% Triton X-100 treatment, and the 60% Percoll:2.5 M sucrose gradient separation. Before Tn5p transposition, the isolated nuclei were examined under a microscope to check their morphology (**Figure 7**). From four independent analyses, the average percentage of intact nuclei was 89.08% with an S.D. of 1.85.

### 4.2 Optimization of nuclei number and Tn5p transposition efficiency

In previous studies of maize ATAC-seq, 50,000 nuclei are commonly used (Lu et al., 2019; Ricci et al., 2019; Crisp et al., 2020). Several Arabidopsis ATAC-seq studies have shown that a variable number of nuclei, ranging from 500–50,000, were used in their experiments (Buenrostro et al., 2013; Gray et al., 2017). To evaluate the appropriate number of maize nuclei for one reaction in our protocol, we tested 500, 2,500, 5,000, and 50,000 nuclei and used genomic DNA as a control. After the transposition reaction, DNA fragments were purified using PCR purification kit for the next amplification step. During the first round of PCR amplification, indexing barcodes were added onto each transposed DNA fragment. After five cycles of PCR, we paused the reaction and took a small aliquot (1 μl) of the PCR product for a qPCR assay to estimate the relative amount of effective DNA template. Higher amounts of DNA template detected by qPCR (i.e. a lower N number) indicate more successful transposition events. This should correlate with the initial number of nuclei used in the reaction.

**Figure 8:**
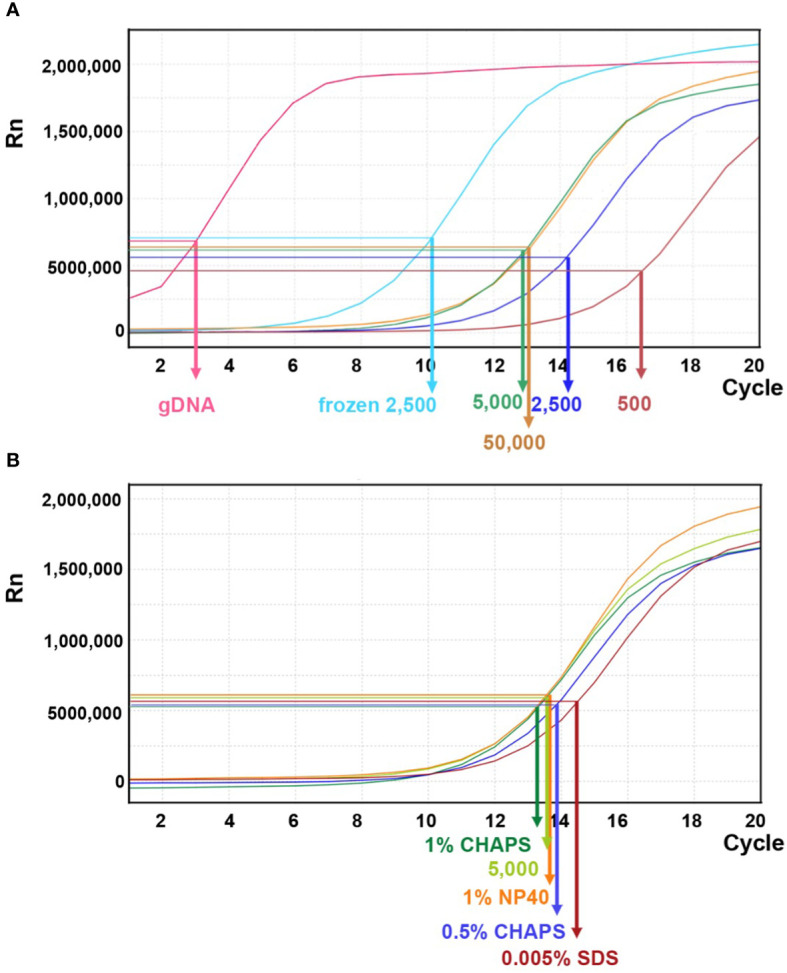
Linear amplification plot of quantitative PCR analysis for different transposition reactions. **(A)** The linear amplification plot versus PCR cycles shows that reactions with 500, 2,500, or 5,000 fresh nuclei (dark brown, dark blue, and green lines, respectively) positively correlate with nuclei numbers. However, the reaction with 50,000 fresh nuclei did not result in an improved outcome. When 2,500 frozen nuclei (light blue line) were used as input, the amplification plot showed a significant increase of DNA template. Genomic DNA (gDNA) serves as a control for the transposition reaction. The arrows indicate the N cycle number of each sample, corresponding to one-third of the maximum signal intensity. **(B)** Quantitative PCR analysis for transposition reactions with different mild detergents using 5,000 fresh nuclei.

As shown in **Figure 8A**, qPCR amplification plots from transposition reactions with 500 and 5,000 nuclei showed positive correlations with effective DNA template. However, reactions with 50,000 nuclei did not further increase effective transposition events. In contrast, the naked genomic DNA exhibited a steep amplification curve, suggesting that free DNA is much more easily targeted by the Tn5p. Moreover, we tested whether using frozen nuclei could enhance transposition events (Corces et al., 2017). We froze approximately 2,500 purified nuclei at -20°C for 30 minutes before proceeding with the transposition reaction. In comparison with 2,500 fresh (non-frozen) nuclei, qPCR analysis revealed a notable increase in transposed DNA fragments within the frozen samples, even exceeding the numbers generated in the reaction using 50,000 nuclei (**Figure 8A**). We reasoned that the freezing process may damage nuclei and chromatin structures, thus more susceptible to Tn5p transposition. In addition, we tested whether a mild detergent in the transposition reaction could increase nuclear permeability for Tn5p transposition. Our results showed that SDS, CHAPS and NP40 did not result in significantly improved outcomes (**Figure 8B**).

### 4.3 Determination of PCR cycle number for amplifying the NGS library

In our libraries of 2,500 and 5,000 fresh maize nuclei, we estimated N cycles of 14 and 13, respectively, based on the cycle number reaching one-third of the maximum value in our qPCR plots (**Table 1**; **Figure 8**). The fact that these N cycle numbers are higher than we expected may suggest that the number of tagged fragments in maize is lower than achieved for other organisms, which may reflect that the maize genome contains a smaller proportion of open chromatin (Lu et al., 2017; Bajic et al., 2018; Maher et al., 2018) or alternatively reflect the less potential complexity due to fewer nuclei. Indeed, a previous study using MNase-seq showed that only a small portion (<1%) of the maize genome resides in open chromatin (Rodgers-Melnick et al., 2016) though MNase-seq is not fully comparable with ATAC-seq. To avoid excessive duplication and PCR bias, we tested the secondary PCR using a reduced number of additional cycles (four fewer than initially estimated). Accordingly, we generated sequencing libraries using 10 and 9 cycles for the 2,500 and 5,000 nuclei samples, respectively, so that the DNA concentrations of the libraries were sufficient for sequencing with limited duplicates (**Table 1**).

**Table 1:** PCR cycles and DNA concentrations of ATAC-seq libraries.

Sample	Treatment	Predicted N cycle	Additional cycles of 2nd PCR	Library concentration (ng/ul)	Total DNA (ng)
50 ng gDNA	NA	3	3	19.50	390
500 nuclei	fresh	16	13	1.97	39
5,000 nuclei	fresh	13	9	1.96	39
50,000 nuclei	fresh	13	9	3.52	70
5,000 nuclei	0.5% CHAPS	14	10	2.02	40
5,000 nuclei	1% CHAPS	14	10	2.44	49
5,000 nuclei	1% NP40	14	10	2.24	45
5,000 nuclei	0.005% SDS	14	10	1.38	28
2,500 nuclei	fresh	14	10	1.51	30
2,500 nuclei	frozen	10	8	8.68	174

### 4.4 Library quality assessment

**Figure 9:**
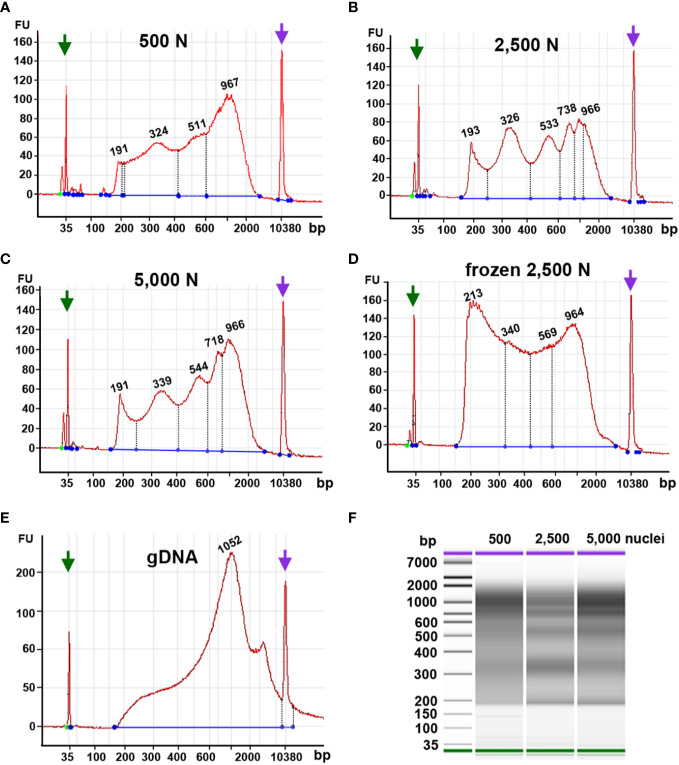
Fragment size distribution of ATAC-seq libraries determined by an Agilent 2100 Bioanalyzer. The X-axis represents the product size in base pairs and the Y-axis is the arbitrary fluorescence intensity (FU). **(A–E)** Fragment size distribution of samples with **(A)** 500 fresh nuclei (500 N), **(B)** 2,500 fresh nuclei (2,500 N), **(C)** 5,000 fresh nuclei (5,000 N), **(D)** 2,500 frozen nuclei, or **(E)** 50 ng genomic DNA (gDNA). Numbers indicate fragment sizes of peaks, which may represent mono- or multiple nucleosomes in the 2,500 nuclei and 5,000 nuclei samples. Purple and green arrows represent upper and lower size markers, respectively. **(F)** A gel-like picture of the fragment size distributions of ATAC-seq libraries generated from 500, 2,500, or 5,000 fresh nuclei.

Tn5p may insert open chromatin regions loosely packed chromatin, which gives rise to transposed DNA fragments with lengths corresponding to mono- or multiple nucleosomes. Thus, a successful ATAC-seq library should exhibit a pattern of fragment size periodicity with intervals of around 200 bp. Additionally, transposed DNA fragments can result from two insertions within nucleosome-free DNA regions. In contrast, if chromatin structures are damaged or perturbed, random transposition can result in a library with various DNA fragment lengths lacking a consistent periodicity. Our Bioanalyzer analysis showed that using 2,500 or 5,000 fresh maize nuclei as input yielded fragment size distributions with the expected ATAC-seq characteristics, i.e., size intervals of ~200 bp (**Figures 9B, C**). The characteristic periodicity is a measurement to evaluate the Tn5p insertion. Although the fragment periodicity of the library may not guarantee the quality of the sequencing data, this serves as a preliminary indicator before sequencing. The reaction using 500 nuclei exhibited a less pronounced periodicity of peak, implying that Tn5p may become oversaturated in reactions with only 500 nuclei (**Figure 9A**).

Although our qPCR results suggested that frozen nuclei resulted in more efficient Tn5p transposition (**Figure 9A**), our Bioanalyzer data revealed that the frozen nuclei sample lacked a clear pattern of periodicity in fragment sizes (**Figure 9D**), suggesting that the chromatin structure may be disrupted by freezing or thawing. While in Arabidopsis ATAC-seq has been successfully performed using frozen samples, our result (**Figure 9**) supports that fresh maize samples showed a better periodic pattern than the maize nuclei frozen in liquid nitrogen, suggesting that frozen samples may not be suitable for maize ATAC-seq experiments. Similarly, Bioanalyzer data indicated that transposed fragments from the naked genomic DNA completely lack size periodicity (**Figure 9E**).

Taken together, these results suggest that 2,500 and 5,000 fresh maize nuclei represent a good starting material for ATAC-seq. In addition, maize ATAC-seq libraries can be generated successfully with an N cycle number lower than typically recommended, as long as the fragment length distribution indicated by Bioanalyzer analysis exhibits the desired characteristic periodicity.

### 4.5 Validation of ATAC-seq

**Figure 10:**
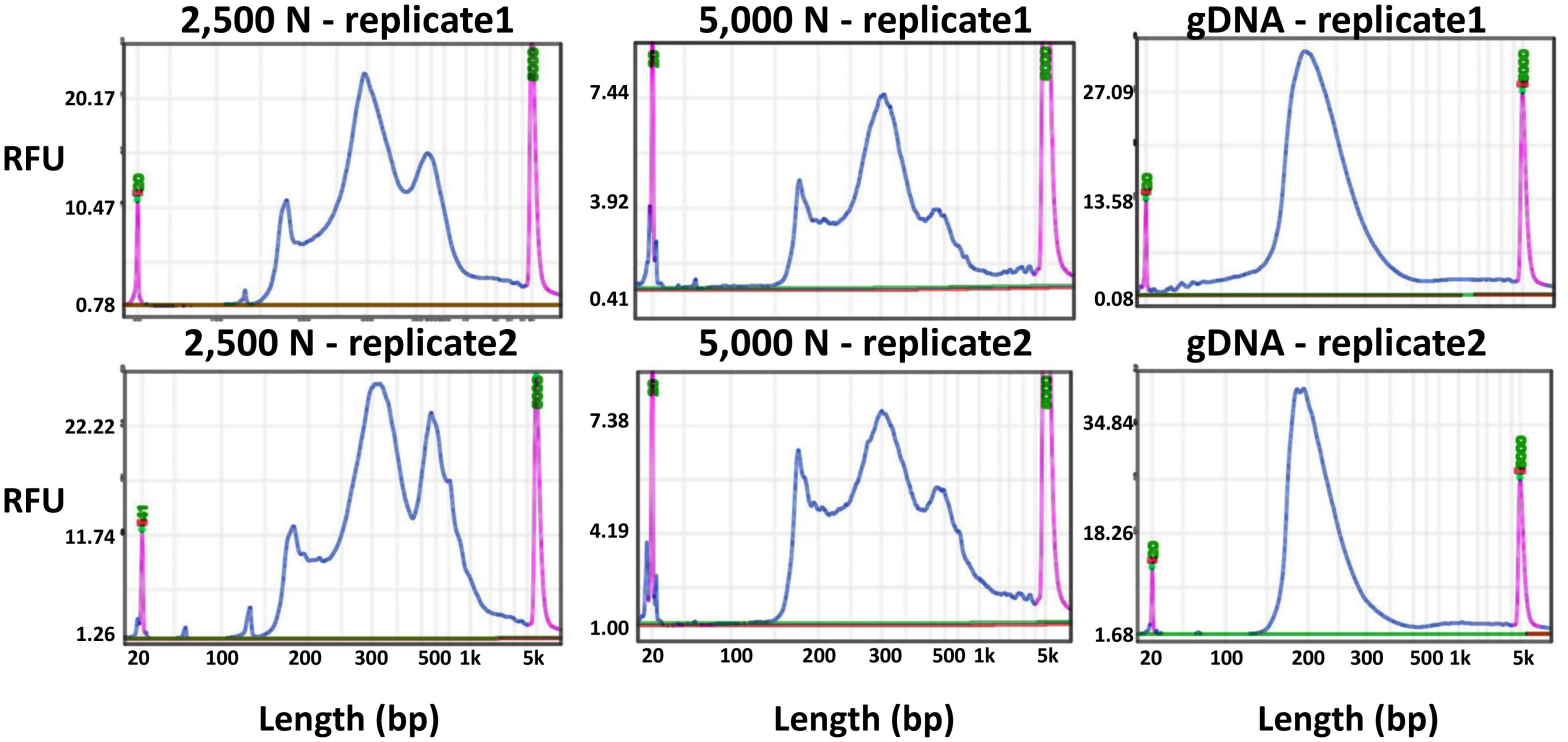
Fragment size distribution of ATAC-seq libraries determined by a BiOptic Qsep400 before NGS sequencing. The X-axis represents the product size in base pairs and the Y-axis is the relative fluorescence unit (RFU). Purple peaks indicate size markers.

**Figure 11:**
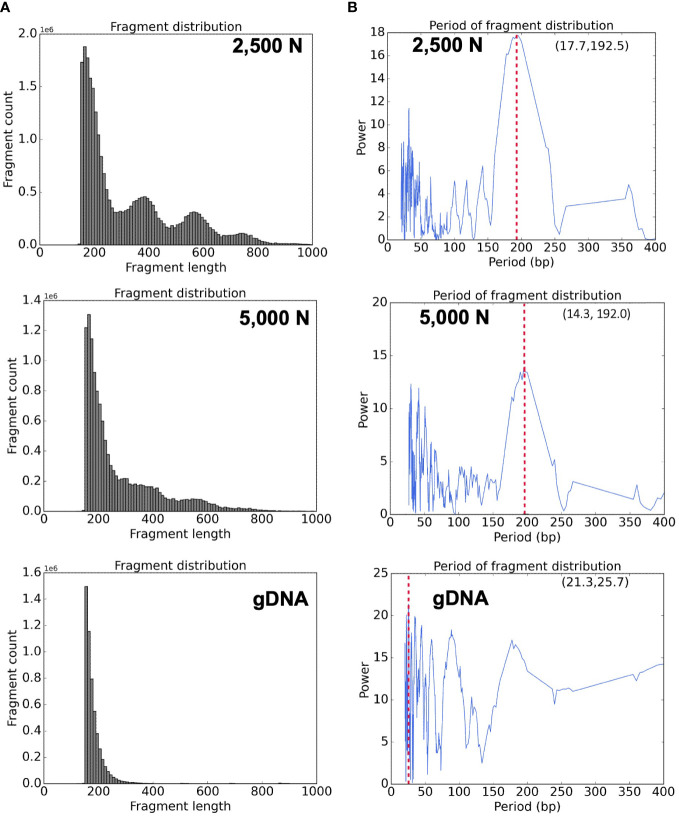
Bioinformatic analyses of ATAC-seq library reads. Fragment size distribution of the **(A)** top: 2,500-nuclei library, middle: 5,000-nuclei library, and bottom: gDNA control. The period of fragment length distribution using a fast Fourier transform (FFT) algorithm for the **(B)** top: 2,500-nuclei library, middle: 5,000-nuclei library, and bottom: gDNA control. The red dashed lines in the graph represent the peaks in the period of fragment length distribution.

**Figure 12:**
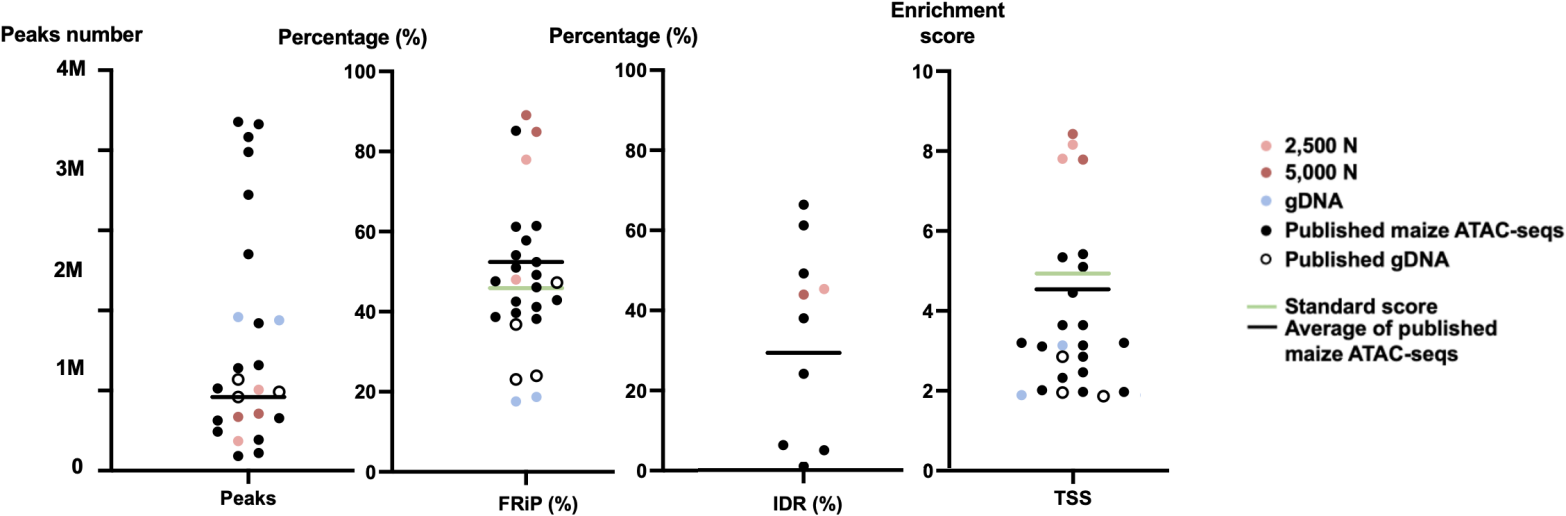
The quality metrics of 2,500-nuclei library, 5,000-nuclei library, published maize ATAC-seq libraries and their matched gDNA control. Four new generated ATAC-seq libraries and 16 published ATAC-seq libraries from six seedlings, six leaves, and two ears, six roots, as well as six matched gDNA are included in the comparison. The accession numbers of the included published ATAC-seq libraries are SRR12321693, SRR12321694, SRR12321695, SRR7889829, SRR7889830, SRR7889827, SRR7889828, SRR7889831, SRR7889832, SRR7904001, SRR7904002, SRR7904003, SRR13920264, SRR13920265, SRR6761057, SRR6761058, SRR6761060, SRR6761061, and SRR11955290. The green lines indicate the ENCODE standards for the metrics and the black lines are the average values for the published maize ATAC-seq libraries (excluding gDNA) of each metric. IDR, irreproducible discovery rate; FRiP, fraction of reads in peaks; TSS, transcription start site; 2,500 N, 2,500-nuclei library; 5,000 N, 5,000-nuclei library; gDNA, genomic DNA.

**Figure 13:**
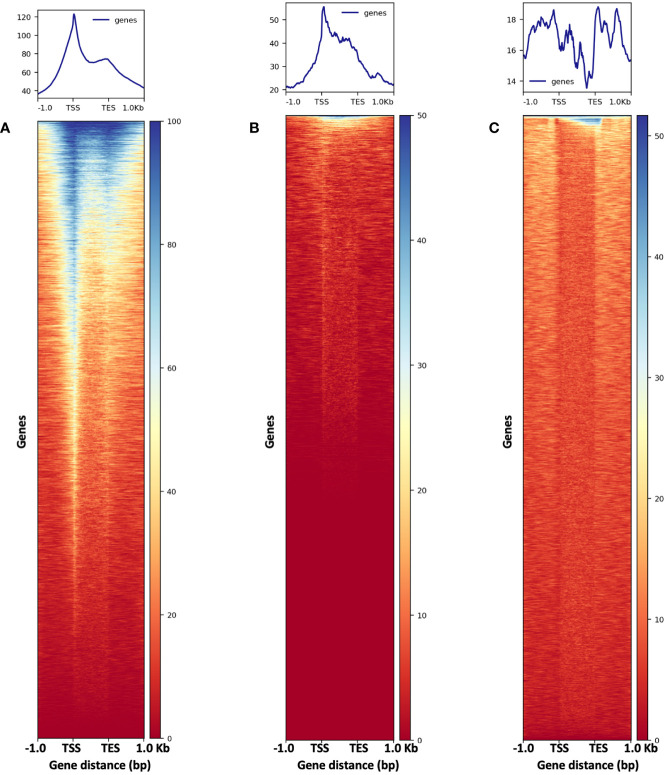
The metaplots (top) and heatmaps (bottom) illustrate read abundance around maize genes. ATAC-seq libraries from **(A)** 2,500-nuclei and **(B)** 5,000-nuclei showing peaks at TSS, in contrast to results from gDNA control **(C)**. TSS, transcription start site; TES, transcription end sites.

**Figure 14:**
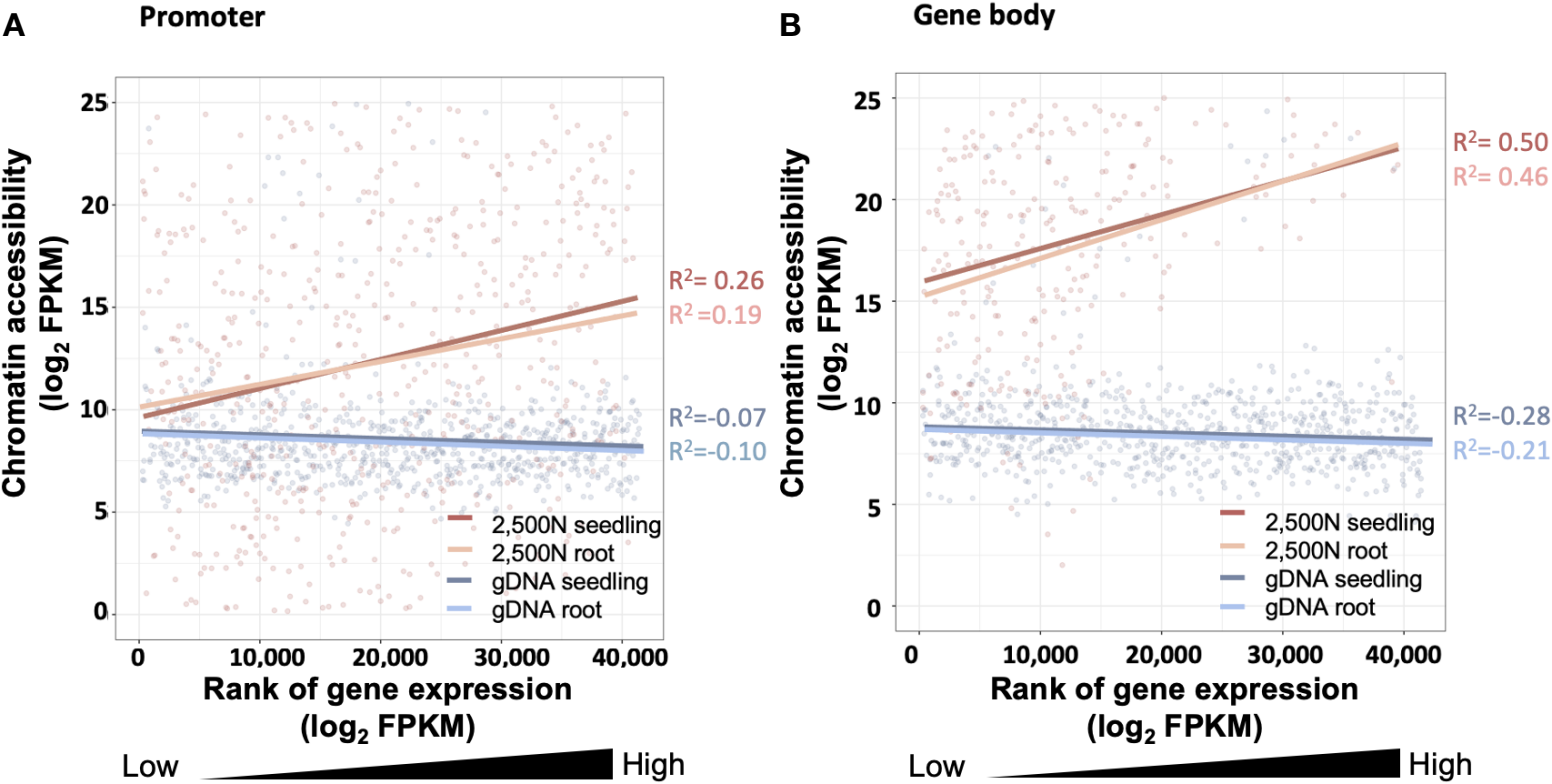
The correlation between chromatin accessibility and transcript abundance for the 2,500-nuclei library and gDNA control at the region of **(A)** promoter and **(B)** gene body. The x-axis is the rank of transcript abundance (log2FPKM) and the y-axis represents the chromatin accessibility analyzed from our ATAC-seq data. The lines with different colors are linear regressions of correlations for ATAC-seq libraries with seedling or root (accession number SRR7548392 and SRR2043190) transcriptome data. The coefficient of correlation is tested by Spearman correlation. FPKM, Fragments Per Kilobase Million.

To evaluate the results of our ATAC-seq protocols, we sequenced ATAC-seq libraries constructed from fresh 2,500 nuclei, 5,000 maize nuclei (two optimized nuclei conditions), and genomic DNA isolated from maize seedlings as a control, each with two biological replicates (**Figure 10**). We assessed our libraries using three methods: (1) analysis of fragment size distribution by plotting histograms to confirm the hallmark of the characteristic size periodicity of ATAC-seq libraries (Buenrostro et al., 2013) (**Figures 10**, **11**); (2) calculation of three standard metrics of ATAC-seq to assess our libraries’ quality (**Figures 12**, **13**), and (3) correlation analysis with maize RNA-seq data from seedling and root tissues (**Figure 14**).

High quality ATAC-seq data is expected to display a clear fragment size periodicity of approximately 200 bp (Buenrostro et al., 2013), a pattern presumably resulting from two Tn5 transposase insertions flanking single or multiple nucleosomes. To compare fragment size distributions, we aligned the paired-end reads to the maize reference genome (AGPv4) using Bowtie2 as described in step 3.2.2 (Langmead and Salzberg, 2012), and then plotted read frequencies against fragment size. As shown in the top and middle graphs of **Figure 11A**, both libraries generated from 2,500 and 5,000 nuclei exhibited clear size periodicity, indicating successful ATAC-seq libraries. The fast Fourier transform (FFT) analysis (**Figure 11B**) confirmed that the periodicity of the fragment length distribution was approximately 192 bp, similar to the expected length of two Tn5p insertions spanning mono-nucleosome protected sequences. In contrast, the library generated from naked genomic DNA lacked the distinct size periodicity (bottom of **Figures 11A, B**), indicating various fragment lengths were derived from random Tn5p insertions into genomic DNA.

To assess the quality of our ATAC-seq libraries, we examined three established metrics commonly applied in ATAC-seq library evaluation (Landt et al., 2012; ENCODE, 2017, 2020; Schmitz et al., 2022). First, the enrichment analysis at Transcription Start Sites (TSS) +/- 1kb is a useful method to validate the efficacy of ATAC-seq in identifying open chromatin in regulatory regions. The fraction of reads in peaks (FRiP) metric assesses the proportion of all mapped reads located in peak regions, indicating the signal-to-noise ratio. Additionally, the irreproducible discovery rate (IDR) is used to calculate the number of irreproducible peaks between replicates (Li et al., 2011) (**Figure 12**). For these metrics, we referenced the ENCODE ATAC-seq standards (ENCODE, 2017, 2020) and guidelines from Schmitz (Schmitz et al., 2022), which suggest that for maize, the FRiP score should exceed 45%, and the TSS enrichment value should be no less than 5. In our analysis, we included 16 published maize ATAC-seq libraries for comparison. Our libraries of 2,500 and 5,000 nuclei maintain above-standard scores and either meet or exceed the averages of published maize ATAC-seq datasets (**Figure 12**). Moreover, we profiled the abundance of ATAC-seq reads around genes. Notably, in the libraries derived from 2,500 and 5,000 nuclei, there is a peak in read abundance at TSS sites (**Figures 13A, B**), which is apparently different from the library generated from naked gDNA control (**Figure 13C**).

Since gene expression is partially affected by chromatin accessibility (Hsu et al., 2017; Chang et al., 2018), examining the association between these two factors serves as a valuable assessment for the ATAC-seq data generated using our protocol. As shown in **Figure 14A**, chromatin accessibility analyzed from our 2,500-nuclei library is positively correlated to RNA transcript abundance obtained from maize seeding and root samples. Interestingly, we observed a slightly stronger correlation between chromatin accessibility in promoter regions with the transcriptome of seedling sample compared to roots, likely reflecting that our ATAC-seq libraries were generated from seedlings (**Figure 14A**).

The correlation analysis in promoter and gene body regions (**Figures 14A, B**) exhibit a similar pattern, suggesting that the open chromatin in promoter regions and gene body are both associated with higher transcript abundance. In contrast, the gDNA ATAC-seq control showed that chromatin accessibility at the promoter and gene body regions exhibited no correlation to gene expression levels.

To have a better sense of the ATAC-seq data from 2500 or 5000 maize nuclei, we showed four genes (lipoxygenases 10, S-adenosyl methionine decarboxylase 2, plasma-membrane H+ATPase2, and nudix hydroxylase 4), including their expression levels and ATAC-seq abundance in our 2500 N and 5000 N libraries using a genome browser (**Supplementary Figure 1**). These genes, or their family members, have been linked to plant defense and early development (Suzuki and Hirasawa, 1980; Christensen et al., 2013; Liu et al., 2022), suggesting that their open state may enhance gene transcription, leading to improved development and adaptation. We generated peaks distribution across genomic features using ChIPSeeker and ATACgraph. Both tools consistently indicated that peaks in our maize ATAC-seqs occur in promoters, gene body, and intergenic regions (**Supplementary Figure 2**).

## 5 Discussion

The most critical step in ATAC-seq protocols is the Tn5p transposition reaction, during which the Tn5p fragments the DNA *in vivo* and tags it with unique Illumina library adaptors. Open chromatin regions have a higher probability of being targeted by Tn5p during the reaction, which is influenced by several critical factors.

First, high purity of nuclei is important, as the Tn5p targets not only genomic DNA, but also mitochondrial and chloroplast DNA. Therefore, it is better to minimize the presence of organelles. In addition, an effective reaction requires Tn5p to be able to attack chromatin efficiently, so the elimination of cell walls and cell debris is necessary to facilitate the accessibility. Second, the native chromatin conformation must be preserved during isolation, as damaged nuclei and disordered chromatin structure could result in distortive results. Last, the ratio of transposase to the number of nuclei should be optimized based on genome size and percentage of open chromatin (i.e., frequency of accessible regions). After an effective and efficient transposition reaction, the resulting fragments tagged with adapters are subjected to two rounds of PCR to generate an ATAC-seq library. In the first round of PCR, these fragments are amplified only by five PCR cycles, during which distinct sequencing barcodes are added. A fraction of this first PCR product is then subjected to quantitative PCR (qPCR) to estimate the relative amount of successfully tagged DNA fragments and then to determine the optimal amplification cycle number for the second round of PCR. Under favorable conditions, an ATAC-seq library is ready for sequencing, when it contains sufficient DNA with appropriate sequence complexity after the second PCR. Since NGS is relatively expensive, applying a proper method for assessing library quality before sequencing can potentially reduce the sequencing expense.

In our testing design, we found that liquid nitrogen-based homogenization is not suitable for maize ATAC-seq analysis, so we instead implement manual chopping for nucleus extraction. This protocol is designed for fresh plant materials using manual chopping, followed by 1% Triton X-100 treatment and 60% Percoll:2.5 M sucrose gradient separation for nucleus extraction. If the materials are stored at −80°C (or in liquid nitrogen) or incorrect concentration of extraction buffer are used, it will lead to a high amount of tissue debris, which can interfere with isolation of intact nuclei.

To conclude, we demonstrated here a protocol that only required a small amount of nuclei without any cell sorting process to identify the open chromatin regions in the plant genome. This strategy shows reliable results even compared to other published maize ATAC-seq libraries. Moreover, this method may be adaptable to other plant species, including crops, thereby contributing to research in plant epigenomics and agriculture.

## Data availability statement

The data presented in the study are deposited in the NCBI repository, accession number GSE252638 (https://www.ncbi.nlm.nih.gov/geo/query/acc.cgi?acc=GSE252638).

## Author contributions

J-WAH: Formal Analysis, Investigation, Validation, Writing – original draft, Writing – review & editing. P-YL: Methodology, Writing – review & editing, Investigation, Software, Validation, Visualization, Writing – original draft. C-TW: Investigation, Methodology, Writing – original draft. Y-JL: Investigation, Methodology, Writing – original draft. PC: Investigation, Methodology, Writing – original draft. RJ-HL: Investigation, Methodology, Writing – original draft. P-YC: Methodology, Conceptualization, Funding acquisition, Resources, Supervision, Writing – review & editing. C-JRW: Conceptualization, Data curation, Methodology, Supervision, Validation, Writing – review & editing.
