# Supplementary material for: Establishing an optimized ATAC-seq protocol for the maize
Source: Front Plant Sci. 2024 May 28;15:1370618. doi: 10.3389/fpls.2024.1370618 (PMC11165127; doi:10.3389/fpls.2024.1370618)
Supplement: Supplementary file 1 [file DataSheet_1.pdf]

Supplementary Material

Supplementary Table

Supplementary Table 1. The summary of RNA-seq data of samples.

Tissue	Raw reads (bp)	Clean reads (bp)	Mapped reads (bp)	Mappability (%)
Seedling	12,931,365	10,678,682	10,465,095	89.92
Root	8,085,432	8,065,410	7,704,543	95.53

Supplementary Table 2. The summary of ATAC-seq data of samples.

Sample	Read number				Mappability (%)	Percent mapped to		Duplicated (%)
	Raw	After duplicate removal	After adapter trimming	Uniquely Mapped		Mitochondrial (%)	Plastid (%)	
2,500 N	719M	262M	224M	222M	99.14	3.05	1.92	63.63
	745M	220M	188M	186M	98.92	1.29	1.53	70.56
5,000 N	594M	186M	163M	161M	99.23	3.15	3.38	68.71
	589M	192M	170M	169M	99.18	3.89	3.43	67.36
gDNA	349M	326M	313M	312M	99.73	0.61	1.45	6.62
	664M	611M	590M	588M	99.73	0.58	1.37	7.84

Supplementary Figure

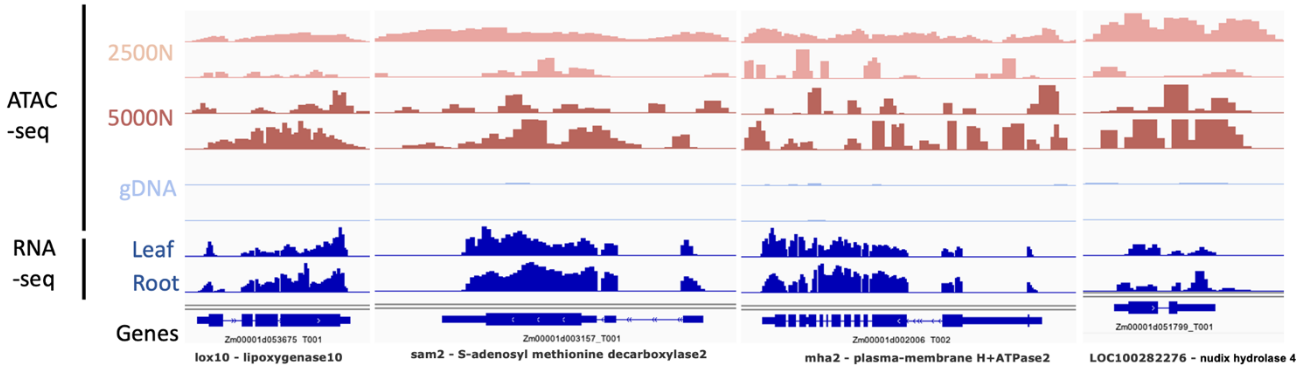

Supplementary Figure 1. Screenshots of four genes with high gene expression levels and ATAC-seq abundance in our 2,500 N and 5,000 N samples, but not in gDNA.

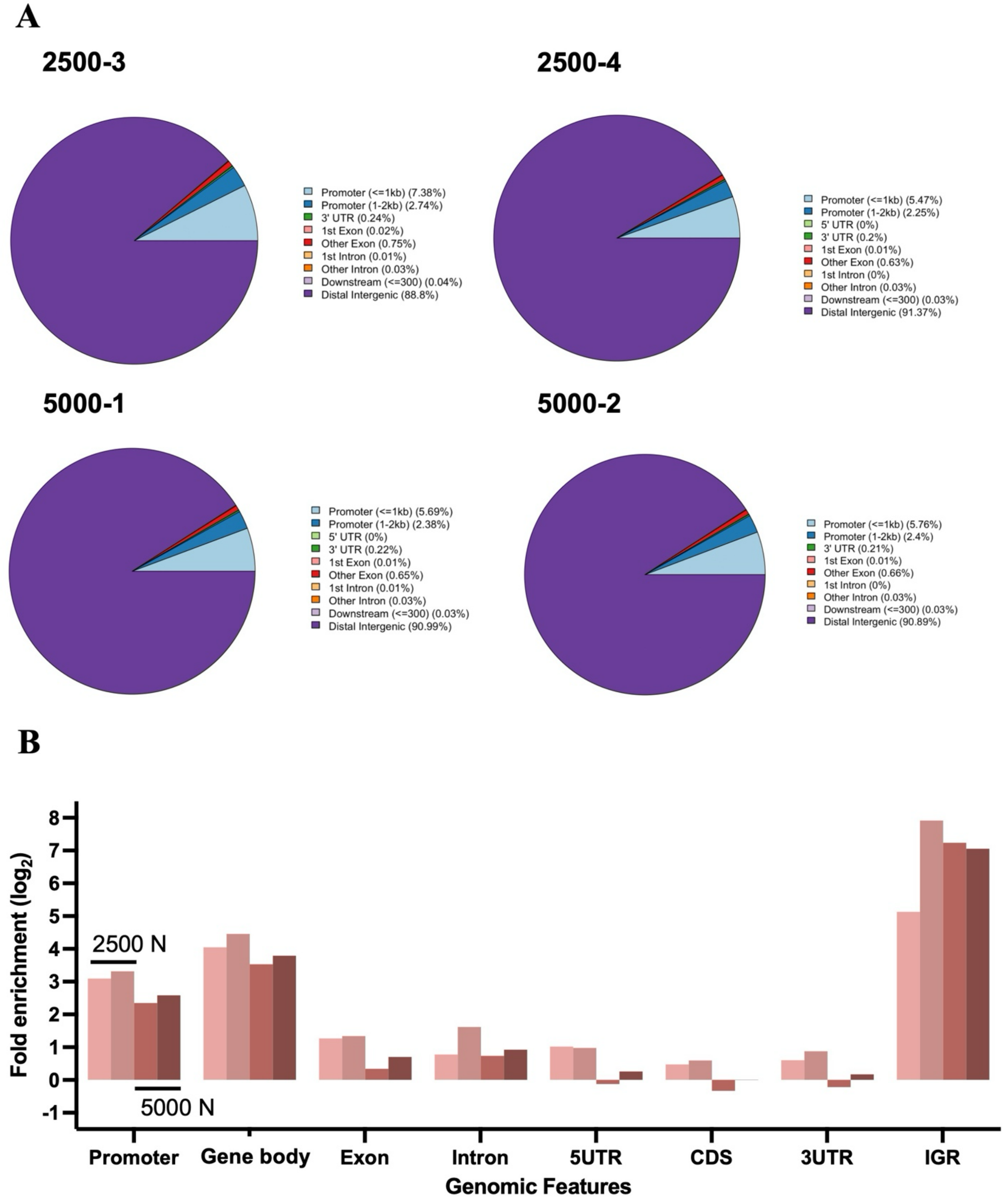

Supplementary Figure 2. Peaks distribution across genomic features from ChIPSeeker (A) and ATACgraph (B).
